# Photocatalytic Degradation of Ciprofloxacin by GO/ZnO/Ag Composite Materials

**DOI:** 10.3390/nano15050383

**Published:** 2025-03-01

**Authors:** Haonan Chi, Pan Cao, Qi Shi, Chaoyu Song, Yuguang Lv, Tai Peng

**Affiliations:** 1 School of Materials Science and Engineering, Jiamusi University, Jiamusi 154000, China; 2 College of Pharmacy, Jiamusi University, Jiamusi 154000, China; 3 School of Chemistry and Chemical Engineering, Shanghai Jiao Tong University, Shanghai 200240, China

**Keywords:** silver, zinc oxide, graphene oxide, photocatalytic degradation, ciprofloxacin, ultraviolet rays

## Abstract

This study synthesized graphene oxide (GO)/zinc oxide (ZnO)/silver (Ag) composite materials and investigated their photocatalytic degradation performance for ciprofloxacin (CIP) under visible light irradiation. GO/ZnO/Ag composites with different ratios were prepared via an impregnation and chemical reduction method and characterized using X-ray diffraction (XRD), scanning electron microscopy (SEM), Fourier-transform infrared spectroscopy (FT-IR), and X-ray photoelectron spectroscopy (XPS). The results demonstrated that under optimal conditions (20 mg/L CIP concentration, 15 mg catalyst dosage, GO/ZnO-3%/Ag-doping ratio, and pH 5), the GO/ZnO/Ag composite exhibited the highest photocatalytic activity, achieving a maximum degradation rate of 82.13%. This catalyst effectively degraded ciprofloxacin under light irradiation, showing promising potential for water purification applications.

## 1. Introduction

Water resources are the basic resources for maintaining the sound operation of ecological systems and influencing the sustainable development of society and economy. China’s per capita water resources amount to only 2200 cubic meters, accounting for only one-fourth of the world’s average level, making it a country with scarce water resources. With the development of the industrialization process, the problem of water resource pollution has become increasingly serious, and the contradiction between the supply and demand of water resources in China has become more acute. Therefore, solving the problem of water pollution and improving the water ecological environment are of particular importance for enhancing the utilization rate of water resources [1]. In recent years, due to the widespread use of antibiotics, mixtures of antibiotics and their metabolites have been continuously released into the water environment through different pathways, and antibiotics in the equivalent range of ng/L-μg/L have been detected in sewage, surface water, and groundwater on a global scale. Research data indicate that the presence of antibiotics in the water environment can disrupt the ecological balance, promote the spread of antibiotic resistance genes, pose risks to water quality, and cause harm to human health through the bioaccumulation effect [2]. The types of antibiotics detected in water bodies mainly include sulfonamides, tetracyclines, fluoroquinolones, macrolides, etc. Fluoroquinolone antibiotics are one of the broad-spectrum antibacterial drugs with the widest application range and the best application effect, targeting the deoxyribonucleic acid of bacteria and having inhibitory and inactivating effects on various bacteria. Among them, ciprofloxacin, as a second-generation fluoroquinolone antibiotic, is widely used in the treatment of various bacterial infections due to its excellent tissue permeability, good bioavailability, and fewer side effects, resulting in the detection of ciprofloxacin in various aquatic environments [3,4]. Since traditional physicochemical and biological treatment methods cannot completely remove ciprofloxacin at low concentrations, seeking a green, efficient, and simple method to remove trace amounts of ciprofloxacin from water has become a research hotspot both at home and abroad [5].

In the photocatalytic process, the photocatalyst generates electron–hole pairs under illumination conditions. The electrons and holes react with oxygen and water adsorbed on the catalyst surface, respectively, to generate reactive oxygen species with strong oxidizing ability, such as hydroxyl radicals (·OH) and superoxide radicals (·O_2_^−^). These reactive oxygen species can oxidize and decompose organic pollutants into harmless substances such as carbon dioxide, water, and inorganic ions [6]. However, wide-bandgap semiconductors only absorb ultraviolet light and have low utilization rates of visible light. The high efficiency of the photogenerated electron–hole (e-h^+^) pair complex hinders the rapid development and practical application of wide-bandgap semiconductors. Therefore, the development of efficient photocatalytic degradation materials is very important [7].

Among many photocatalysts, zinc oxide (ZnO) has the advantages of good photocatalytic activity, stability, non-toxicity, and low cost, which is beneficial for industrial applications [8]. ZnO has been fabricated into nanoparticles, nanosheets, nanowires, nanorods, and nanobelts. Among them, one-dimensional (1-D) nanostructures have uniform shapes and efficient separation and transport of photogenerated electrons. Moreover, it is a wide-bandgap semiconductor material with a bandgap of 3.37 eV. It can generate electron–hole pairs under ultraviolet light irradiation and thus exhibit photocatalytic activity. However, pure ZnO also has some shortcomings, such as easy recombination of photogenerated electron–hole pairs and low utilization rate of visible light, which limit its practical application [9]. To improve the photocatalytic performance of ZnO, various methods have been adopted to modify it, such as doping metal ions, combining with other semiconductors, and loading noble metals [10]. One of them is the application of carbon-based materials because they can enhance the charge transfer at the interface between metal oxides and carbon materials. Carbon nanotubes, graphite, graphene, and graphene oxide (GO), when combined with ZnO, have been shown to enhance the photocatalytic efficiency of ZnO. Ahmad et al. reported the synthesis of GO/ZnO/Ag nanocomposites via the solvothermal method [11]. However, the synthesis of graphene is often challenging due to its high impurity content. Despite this, graphene oxide (GO) presents several advantageous properties that make it a highly attractive material. These include its superior mechanical strength, excellent electrical conductivity (which arises from its sp² hybridized carbon orbitals), unique chemical characteristics such as the potential for zero bandgap behavior, an exceptionally high specific surface area, excellent transparency to charge carriers (owing to its single-atom-thick structure), and outstanding thermal conductivity. These remarkable properties render GO a highly promising candidate for various applications, including its integration with ZnO to improve photocatalytic performance [12]. Due to its large specific surface area and two-dimensional planar conjugated structure, graphene oxide (GO) serves as an ideal scaffold for the effective anchoring of various substances. For instance, the incorporation of GO with photocatalysts such as TiO_2_ or WO_3_ has been extensively studied [13]. GO-based photocatalysts can prevent the agglomeration of nanoparticles immobilized on the graphene sheets, thereby increasing the available reaction sites for photocatalytic degradation. This structural feature not only enhances the adsorption capacity but also facilitates the degradation of organic pollutants. As an example, Nasrollahzadeh et al. synthesized graphene oxide/ZnO nanocomposites and employed them as heterogeneous catalysts for the synthesis of a variety of tetrazoles [14,15,16,17]. Additionally, GO-based photocatalysts for organic contaminant removal in aqueous systems offer advantages in terms of ease of separation, which facilitates catalyst recovery and reuse, thus supporting their potential for large-scale industrial applications [18,19,20,21,22].

The excellent electrical conductivity of graphene oxide (GO) facilitates the transfer of photogenerated electrons and effectively suppresses the recombination of electron–hole pairs, thereby enhancing photocatalytic efficiency [23]. Additionally, the functional groups on GO can form chemical bonds with ZnO, which improves the stability of the composite material. Although the concurrent growth of ZnO and reduction in GO may lead to an uneven distribution of ZnO on the GO surface, this remains a viable approach, especially when other materials are introduced to mitigate such issues [24]. Various dopants, such as metals and metal oxides, have been employed to enhance photocatalytic activity by narrowing the bandgap of ZnO [25]. Noble metals, such as silver (Ag), exhibit surface plasmon resonance (SPR) effects. When Ag nanoparticles are integrated into the ZnO surface, the SPR effect under visible light irradiation amplifies the light absorption capabilities of ZnO, thereby improving the generation efficiency of photogenerated carriers [26,27]. Furthermore, Ag can function as an electron trap, promoting the transfer of photogenerated electrons while inhibiting the recombination of electron–hole pairs, which further augments the photocatalytic performance of ZnO [28,29,30]. Consequently, the synthesis of GO/ZnO/Ag composite materials, which combines the unique advantages of Ag, ZnO, and GO, holds significant potential for enhancing the photocatalytic activity of the composite system.

This paper aims to develop a highly efficient and stable photocatalytic material–GO/ZnO/Ag composite material for treating organic pollutants such as ciprofloxacin. Through research on aspects such as the preparation, characterization, and photocatalytic performance of the composite material, the photocatalytic mechanism will be thoroughly explored, providing a theoretical basis and technical support for the application of photocatalytic technology in the field of environmental governance [31]. Furthermore, through the synergistic effects of the nanocomposite material, ciprofloxacin can be efficiently degraded and transformed into non-toxic byproducts, thereby minimizing potential harm to aquatic ecosystems. This process reduces the persistence and accumulation of pollutants in water bodies, contributing to the protection of water resources and overall ecological health. Such an approach aligns with the principles of sustainable development, facilitating the creation of a cleaner and healthier environment. The motivation behind this research holds significant implications for advancing environmental protection efforts and effectively harnessing light energy to address the growing issue of antibiotic pollution in aquatic systems.

## 2. Materials and Methods

### 2.1. Chemicals and Reagents

Silver nitrate, sodium nitrate, zinc acetate, sodium hydroxide, graphite powder, potassium permanganate, concentrated sulfuric acid, hydrogen peroxide, cetyltrimethylammonium bromide (CTAB), ammonia water, N,N-dimethylformamide (DMF), sodium citrate, ethanol, methylene blue, and hydrochloric acid were all analytically pure and purchased from Aladdin Chemical Reagent. Deionized water was used in the experiment.

### 2.2. Synthesis of Graphene Oxide (GO)

A 100 mL three-necked flask was charged with 23 mL of concentrated sulfuric acid, which was sealed and placed in a refrigerator for 30 min. Meanwhile, 0.5 g of graphite powder and 0.25 g of sodium nitrate were added to the flask and stirred in an ice bath at a temperature of 4–7 °C for 30 min, resulting in the formation of a black suspension. Gradually, 3 g of potassium permanganate was introduced to the mixture, and the temperature was raised to 35 °C, maintaining stirring for 2 h. At this stage, the solution turned dark green. Subsequently, 50 mL of deionized water was added dropwise, and the temperature was increased to 95 °C, followed by stirring for an additional hour, resulting in a brown-yellow solution. Then, 100 mL of deionized water was added to the reaction mixture, and stirring was continued at room temperature for 1 h. Following this, 15 mL of 30% hydrogen peroxide was carefully introduced, causing the solution to boil vigorously and produce abundant bubbles. The color transitioned from an earthy brown to a bright golden hue, and the mixture was stirred at room temperature for 2 h. Afterward, the reaction mixture was allowed to stand and precipitate. The supernatant was decanted, and the precipitate was centrifuged and filtered. The solid was washed three times with 5% hydrochloric acid, followed by several washes with deionized water. Finally, the product was dried in a vacuum oven at 60 °C for 6 h to yield graphene oxide (GO).

### 2.3. Synthesis of GO/ZnO Composite Materials

A precise amount of 3.0 g of zinc acetate was weighed and added to a beaker. To this, 50 mL of deionized water, 2.0 mL of hydrogen peroxide (H_2_O_2_), and 0.4 g of cetyltrimethylammonium bromide (CTAB) were sequentially added, with thorough stirring. Next, 4.0 mL of ammonia solution (NH_3_·H_2_O) was added dropwise under vigorous stirring, and the reaction was allowed to proceed for 9 h. Following this, the reaction mixture was allowed to stand to facilitate precipitation, after which the supernatant was decanted. The precipitate was then subjected to centrifugation and filtration. The resulting product was dried in a vacuum oven at 80 °C for 4 h and subsequently calcined in a muffle furnace at 475 °C for 3 h to obtain zinc oxide (ZnO). For the synthesis of the composites, 0.0005 g, 0.0010 g, and 0.0030 g of ZnO were dispersed in 40 mL of deionized water under ultrasonic agitation, labeled as solutions 1, 2, and 3, respectively. An amount of 0.1 g of graphene oxide (GO) was added to these solutions and ultrasonically dispersed for 30 min. The resulting mixtures were transferred to a water bath at 25 °C and stirred magnetically for 4 h to ensure a complete reaction, yielding a suspension. The suspension was then subjected to centrifugation, followed by washing with ethanol. The final products, GO/ZnO-0.5%, GO/ZnO-1%, and GO/ZnO-3%, were obtained after further drying.

### 2.4. Synthesis of GO/ZnO/Ag Composite Materials

Under magnetic stirring, 15.7 mg of silver nitrate was dissolved in 100 mL of deionized water to prepare the solution. To this, 2 mL of a 1% sodium citrate solution was added as a reducing agent. The resulting mixture was then heated to boiling and maintained for 30 min. After the reaction, the solution was cooled to room temperature. The mixture was then transferred to a centrifuge tube. An amount of 50 mL of anhydrous ethanol was added to the tube, and the mixture was centrifuged at 5000 rpm for 10 min. The supernatant was discarded, and the remaining nanoparticles were resuspended in fresh ethanol. This washing step was repeated 3 times to ensure the removal of residual citrate and other soluble impurities. After the final wash, the nanoparticles were collected by centrifugation, and the ethanol was removed. The purified nanoparticles were then dried under vacuum at 60 °C for 12 h to remove any residual solvent.

For the synthesis of the GO/ZnO/Ag composite materials, the process involved using the GO/ZnO composite as a medium for silver nanoparticle deposition. Initially, the GO/ZnO-0.5%, GO/ZnO-1%, and GO/ZnO-3% composites were dispersed in 40 mL of deionized water under ultrasonic agitation, and labeled as Solution 1, Solution 2, and Solution 3, respectively. Once uniform dispersion was achieved, 0.1 mg of silver nanoparticles was added to each solution, which was then ultrasonically dispersed for 30 min. The resulting mixture was transferred to a water bath at 25 °C and continuously stirred magnetically for 4 h to ensure the formation of a stable suspension.

Subsequently, the suspension was transferred to a polytetrafluoroethylene (PTFE) autoclave and heated at 180 °C for 4 h. After cooling to room temperature, the crude product was filtered, and the final product was washed several times with deionized water and absolute ethanol. The product was then dried under vacuum at 60 °C for 12 h to yield the GO/ZnO-0.5%/Ag, GO/ZnO-1%/Ag, and GO/ZnO-3%/Ag composites.

### 2.5. Sample Characterization

Structural and compositional characterization of the samples was carried out using a suite of advanced analytical techniques. X-ray diffraction (XRD) analysis was carried out using a Rigaku 3DMAX-IIIC diffractometer (Rigaku Corporation, Tokyo, Japan) and Cu Kα radiation (λ = 1.5406 Å) to determine the crystal structure and phase composition. Diffraction patterns were acquired in the 2θ range of 10–70° with a scanning rate of 2°/min and a step size of 0.02°. Fourier-transform infrared spectroscopy (FT-IR) measurements were performed on a Nicolet NEXUS-670 spectrometer (Thermo Fisher Scientific Inc., Waltham, MA, USA) equipped with a deuterated sulfated glycine sulfate (DTGS) detector to determine the presence of functional groups and chemical bonds in the sample. Spectra were recorded over a range of 4000–400 cm^−1^ with a resolution of 4 cm^−1^ and a cumulative total of 32 scans per measurement. Morphological characterization was performed using a field emission scanning electron microscope (FE-SEM, Shimadzu JSM-7800F, Shimadzu Corporation, Kyoto, Japan) with an accelerating voltage of 5–15 kV. Prior to imaging, the samples were sputter-coated with a thin layer of gold to enhance electrical conductivity. X-ray photoelectron spectroscopy (XPS) analysis was performed using a Thermo Scientific ESCALAB 250Xi spectrometer (Thermo Fisher Scientific, Norristown, PA, USA) and a monochromatic Al Kα X-ray source (hν = 1486.6 eV) to determine the elemental compositions, chemical states, and bonding configurations. The analysis was performed under ultra-high vacuum conditions (base pressure < 5 × 10^−9^ mbar). The binding energy scale was calibrated against the C 1s peak at 284.6 eV to compensate for any charging effects and to eliminate potential interference from incidental carbon contamination. High-resolution spectra with a pass energy of 20 eV and a step size of 0.1 eV were used for detailed chemical state analysis. The acquired data were processed using appropriate software packages, including MDI Jade 6.5 for XRD analysis, OMNIC for FTIR spectroscopy, ImageJ for SEM/TEM image analysis, and Avantage for XPS data processing. The combination of these complementary characterization techniques provides a comprehensive understanding of the structural, morphological, and chemical properties of the materials under study.

### 2.6. Catalytic Degradation

In order to study the degradation performance of the synthesized composite photocatalyst, CIP aqueous solution was used as the simulated pollutant, and a xenon lamp was used as the visible light source. Take 50 mL of CIP solution and a certain amount of catalyst, stir well, and let stand in the dark environment for 30 min under the stirred state. Then, turn on the light source and keep stirring. An amount of 5 mL supernatant was taken at intervals and the absorbance of the system at the maximum absorption wavelength of 278 nm was measured by ultraviolet spectrophotometer [17]. The wavelength range was 200–800 nm. The solution was placed in a 1 cm colorimetric dish with deionized water as the blank. The degradation rate of organic carbon in the system is shown in the following Equation (1).

(1) $$D=\left(A_{0}-A_{t}\right)/A_{0}\times 100\%$$

where D is the degradation rate (%), *A*_0_ is the initial absorbance value of CIP, and *A_t_* is the absorbance value after t time of CIP degradation.

## 3. Results and Discussion

### 3.1. Surface Morphology Analysis (SEM)

**Figure 1:**
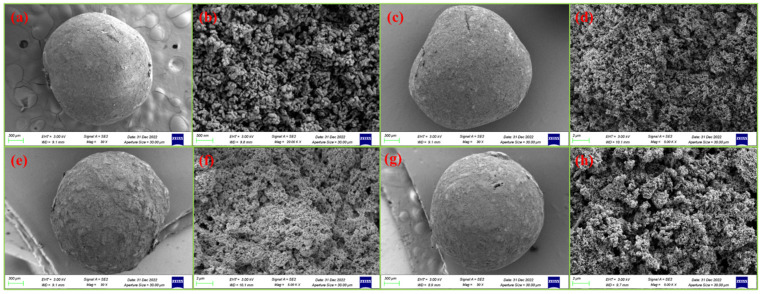
SEM image of (**a**,**b**) GO/ZnO, (**c**,**d**) GO/ZnO-0.5%/Ag, (**e**,**f**) GO/ZnO-1%/Ag, (**g**,**h**) GO/ZnO-3%/Ag nanocomposites.

The morphology of the GO/ZnO, GO/ZnO-0.5%/Ag, GO/ZnO-1%/Ag, and GO/ZnO-3%/Ag nanocomposites was analyzed using scanning electron microscopy (SEM), as illustrated in Figure 1. Panels Figure 1a,b reveal the growth of ZnO, showing the aggregation of graphene sheets along with the formation of spherical ZnO nanoparticles. SEM images further demonstrate that both ZnO and Ag are uniformly distributed and well integrated onto the GO surface, as depicted in panels Figure 1c–h. Notably, the GO/ZnO-3%/Ag composite exhibits the largest surface area and the most intimate interaction with the graphene sheets, which significantly enhances its photocatalytic activity.

### 3.2. X-Ray Photoelectron Spectroscopy (XPS) Analysis

**Figure 2:**
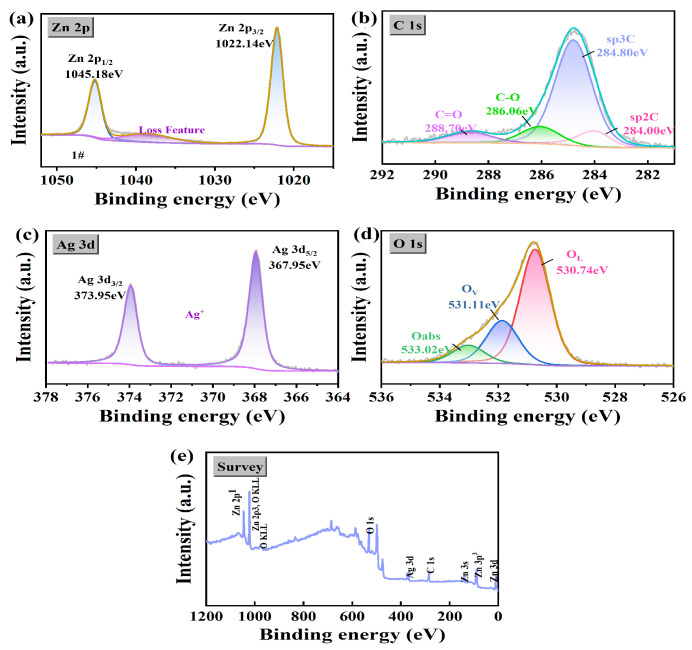
Survey (**e**) and high-resolution X-ray photoelectron spectra of GO/ZnO-3%/Ag: (**a**) Zn2p, (**b**) C 1s, (**c**) Ag 3d, and (**d**) O 1s.

Based on the X-ray photoelectron spectroscopy (XPS) data shown in Figure 2, combined with high-resolution spectroscopic analyses, the elemental compositions and chemical states of the prepared composites were thoroughly investigated.

In Figure 2b, the C 1s spectrum shows four distinct peaks representing different carbon bonding environments. Among them, the peak at 284.00 eV is attributed to sp^2^ hybridized carbon (sp2C), which is a component in graphene oxide (GO), while the higher binding energy peak at 284.80 eV corresponds to sp^3^ hybridized carbon (sp3C), suggesting that the complexes contain a variety of carbon species. The 286.06 eV and 288.70 eV peaks are associated with the C-O and C=O bonds, respectively, indicating the presence of oxidized functional groups on GO, which is typical for GO-based materials [32].

For the sp2C component in Figure 2b, an asymmetric peak shape is presented on the side of the higher binding energy, and this asymmetry can be described by the Doniach–Sunjic function. This function can effectively capture the asymmetric properties of the peaks caused by spin–orbit coupling and core cavity effects. In the analysis process, the spectra were first processed with background subtraction. The Shirley background removal method was used to eliminate the interference of non-featured signals in the spectrogram to ensure the accurate extraction of the target peaks. After background subtraction, the spectra were fitted with a Gaussian–Lorentzian hybrid function, where the asymmetric part of the sp2C was fitted by the Doniach–Sunjic function, and the remaining peaks were fitted by the standard Gaussian or Gaussian–Lorentzian hybrid function, in order to obtain the binding energy positions of the carbon for each chemical state and to gain an in-depth understanding of the distribution of the different carbon species in the composites [33].

The Zn 2p spectrum in Figure 2a shows two characteristic peaks located at 1022.14 eV (Zn 2p_3/2_) and 1045.18 eV (Zn 2p_1/2_). These peaks are consistent with the binding energy of Zn^2+^, indicating that the zinc in the composite is mainly present in the oxidation state of Zn^2+^. In addition, the loss features at higher binding energies are typical of XPS spectra and are usually attributed to the inelastic scattering of photoelectrons.

The Ag 3d spectrum in Figure 2c shows two distinct peaks located at 367.95 eV and 373.95 eV, corresponding to the Ag 3d_5/2_ and Ag 3d_3/2_ electronic lines, respectively. These peaks indicate that silver is mainly present in the composites as Ag^+^, confirming the +1 oxidation state of silver.

The O 1s spectrum in Figure 2d, on the other hand, can be decomposed into three main peaks: the peak at 530.74 eV is attributed to lattice oxygen (OL) in metal oxides, the peak at 531.11 eV is correlated with oxygen vacancies (OV), indicating the presence of oxygen defects in the material, and the peak at 533.02 eV is attributed to surface-adsorbed oxygen (Oabs), which is usually derived from surface-adsorbed hydroxyl or water molecules.

The full spectrum in Figure 2e shows the elemental composition of the composites, confirming the presence of Zn, Ag, O, and C and showing characteristic peaks corresponding to Zn 2p, O 1s, Ag 3d, and C 1s. No other impurities or undesired phases were detected, indicating the high purity of the synthesized composites.

### 3.3. X-Ray Diffraction (XRD) Analysis

**Figure 3:**
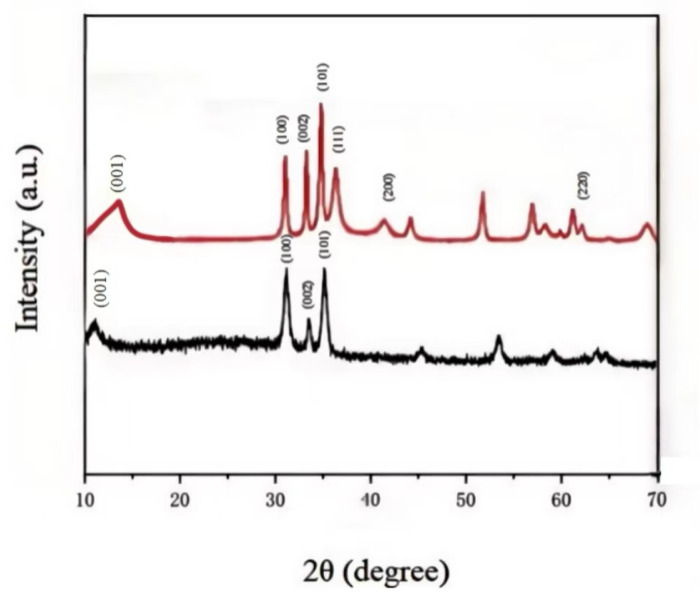
X-ray diffraction (XRD) analysis of GO/ZnO and GO/ZnO-3%/Ag nanocomposites.

In order to check the phase, crystal structure, and purity of the synthesized nanomaterials, X-ray diffraction (XRD) analysis was carried out. The XRD patterns of GO/ZnO and GO/ZnO/Ag nanocomposites are shown in Figure 3. The diffraction peaks of the GO/ZnO composites clearly correspond to the hexagonal sphalerite structure of ZnO. Specifically, the observed peaks at 2θ values of 31.2°, 34.1°, and 35.9° are attributed to the (100), (002), and (101) crystal planes, respectively, in accordance with the JCPDS card no. 36-1451. In particular, the significant intensity of the (101) peak indicates the anisotropic growth of the ZnO crystals and suggests the preferred direction of growth of the microcrystals.

For GO/ZnO/Ag nanocomposites, significant differences are observed when compared to the XRD patterns of GO/ZnO composites. In particular, three additional peaks appeared at 2θ values of 38.1°, 44.3°, and 64.4°, attributed to the (111), (200), and (220) crystal planes, respectively. These peaks are consistent with the crystallographic facets of silver, in accordance with JCPDS card number 04-0783, confirming the successful doping of silver into the composite structure.

### 3.4. FT-IR Analysis

**Figure 4:**
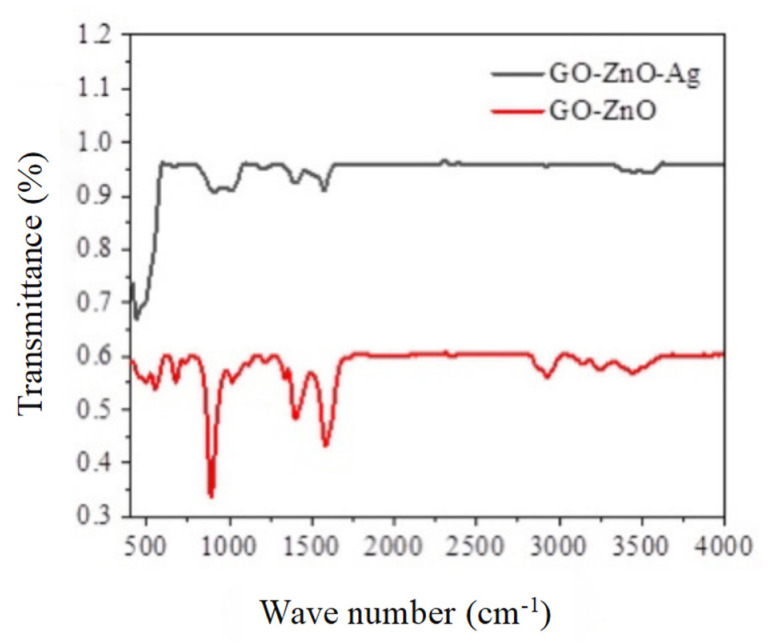
Fourier-transform infrared spectroscopy (FT-IR) spectra of GO/ZnO and GO/ZnO-3%/Ag nanocomposites.

The Fourier-transform infrared (FT-IR) spectra of the GO/ZnO and GO/ZnO/Ag nanocomposites are presented in Figure 4. The characteristic peaks corresponding to the stretching vibrations of oxygen–carbon bonds in graphene oxide are observed at 1108 cm^−1^, 1222 cm^−1^, and 1731 cm^−1^. Additionally, a peak at 1568 cm^−1^ is attributed to C-C bond vibrations, as well as the bending vibrations of epoxy groups and hydroxyl deformation (O-H). A broad absorption band observed at 3454 cm^−1^ corresponds to the hydroxyl (-OH) stretching vibrations of water molecules adsorbed on the surface, while the carboxyl (-COOH) group also contributes to this broad feature [34].

Furthermore, the peak at 450 cm^−1^ is assigned to the Zn-O bond, confirming the formation of ZnO. In the GO/ZnO/Ag nanocomposite, a peak at 1629 cm^−1^ is observed, corresponding to the C-C bond vibration region. Additionally, a peak at 1021 cm^−1^ may be linked to the symmetric and asymmetric stretching vibrations of GO, providing further evidence of the successful incorporation of graphene oxide into the composite material.

### 3.5. Photocatalytic Activity Measurement

**Figure 5:**
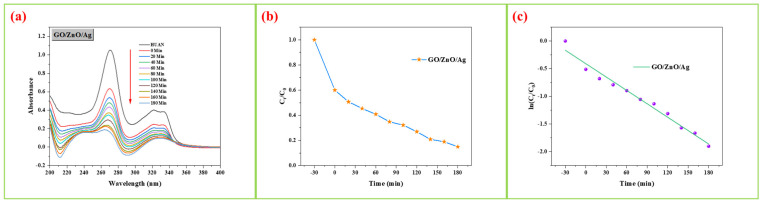
The UV–visible spectra of photocatalytic activity for (**a**) GO/ZnO/Ag nanocomposite; (**b**) UV–visible photocatalytic degradation of CIP; (**c**) UV–visible photocatalytic degradation of CIP kinetics rate constant with GO/ZnO/Ag nanocomposite.

The GO/ZnO/Ag nanocomposite was synthesized using a photocatalytic method and tested for its catalytic degradation of ciprofloxacin under direct sunlight, as depicted in Figure 5a. The photocatalytic activity of the GO/ZnO/Ag nanocomposite was evaluated by monitoring the intensity of the characteristic absorption peak of ciprofloxacin at 278 nm, which allowed for the assessment of degradation efficiency in aqueous solution under sunlight irradiation. The photocatalytic performance is influenced by the material’s ability to absorb light and its electron–hole pair recombination rate. Efficient generation of hydroxyl radicals (·OH) is crucial for effective degradation, which requires an appropriate bandgap energy to match the energy of incident photons. By enhancing charge carrier recombination rates and promoting effective electron transfer, the GO/ZnO/Ag nanocomposite demonstrates significantly improved photocatalytic activity.

During the degradation process, the absorption spectrum of ciprofloxacin evolves, and a strong interaction between ZnO/GO and Ag is observed, leading to the formation of C-O bonds. This bonding reduces the reactivity of the surface oxygen atoms in the composite, enhancing its photostability. The degradation study shows that as the irradiation time increases, the concentration of ciprofloxacin decreases, accompanied by a reduction in the absorption intensity in the UV–visible region. Under direct sunlight, the GO/ZnO/Ag nanocomposite achieved an 82.13% degradation rate of ciprofloxacin, as shown in Figure 5a, demonstrating its high photocatalytic efficiency.

The photocatalytic degradation process of ciprofloxacin using the GO/ZnO/Ag nanocomposite is illustrated in Figure 5b. Typically, semiconductor-based photocatalysis involves the excitation of electrons, leading to the formation of electron–hole pairs, which subsequently participate in chemical reactions. The photogenerated holes migrate to the surface of the particles, where they react with the surrounding medium and ciprofloxacin molecules, producing CO_2_ and H_2_O as by-products.

The kinetics of ciprofloxacin degradation were studied under optimal experimental conditions, including a ciprofloxacin concentration of 20 mg/L, a catalyst dosage of 15 mg, and an adsorption–desorption equilibrium time of 180 min. The degradation follows pseudo-first-order kinetics, as indicated by the linear relationship between ln(C/C_0_) and time, where C represents the ciprofloxacin concentration, and C_0_ is the initial concentration. The apparent first-order rate constant (k_app_) was determined from the equation ln(C_0_/C) = k_app_t, yielding a value of k_app_ = 0.00807 min^−1^ for the GO/ZnO/Ag nanocomposite, as shown in Figure 5c. This result underscores the effective catalytic performance of the nanocomposite in the photocatalytic degradation of ciprofloxacin.

**Figure 6:**
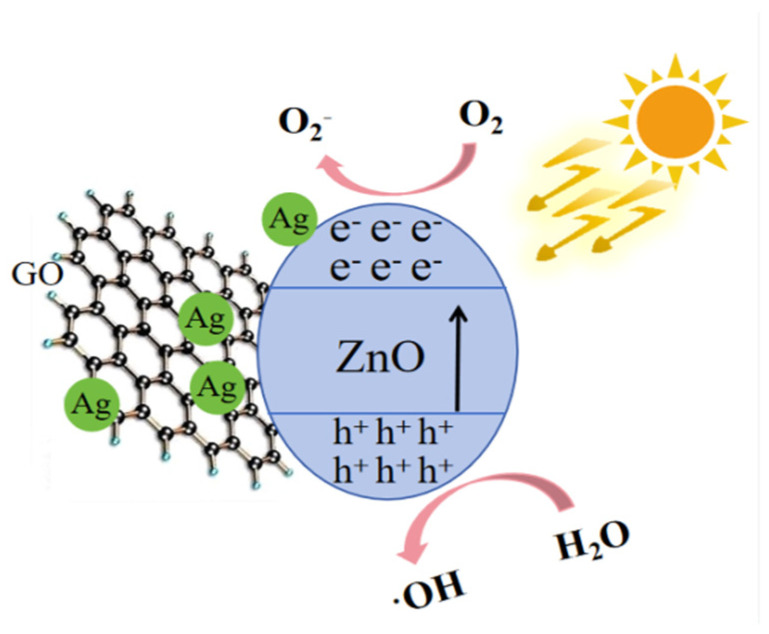
Photocatalytic mechanism diagram.

The photocatalytic degradation mechanism of GO/ZnO/Ag composites involves multiple synergistic effects, as shown in Figure 6. First, ZnO, under UV light irradiation, generates electron–hole pairs (e^−^/h^+^), and its wide bandgap allows efficient absorption of UV light, thereby driving photocatalytic reactions. The generated electrons can rapidly transfer to Ag nanoparticles, while the holes participate in the oxidation of water molecules, producing highly oxidative hydroxyl radicals (•OH). Ag, acting as an “electron sink”, not only enhances the local electric field through surface plasmon resonance (SPR) effects but also effectively prevents the recombination of electrons and holes, thus improving the overall photocatalytic efficiency. Graphene oxide (GO) serves as an excellent electron conduction platform, facilitating the efficient transfer of electrons within the composite material. Moreover, its large surface area aids in the adsorption of pollutants. The synergistic interactions between these three components significantly enhance the generation and transfer efficiency of electrons and active species during the photocatalytic degradation process, thereby boosting the degradation of organic pollutants. Therefore, the superior photocatalytic performance of the GO/ZnO/Ag composite material is primarily attributed to the synergistic enhancement effects of ZnO, Ag, and GO.

## 4. Conclusions

In conclusion, the GO/ZnO/Ag composite materials exhibit excellent photocatalytic activity for the degradation of ciprofloxacin (CIP), with a maximum degradation rate of 82.13% under optimal conditions (20 mg/L CIP concentration, 15 mg catalyst dosage, GO/ZnO-3%/Ag-doping ratio, and pH 5). However, further studies are needed to improve the long-term stability and recyclability of the composite materials. Future research could focus on enhancing the stability of these composites under prolonged exposure to light, evaluating their photocatalytic activity for other environmental pollutants, and exploring scalable synthesis methods. Additionally, a deeper understanding of the photocatalytic degradation mechanism is essential to optimize the performance of the materials.

## Data Availability

The original contributions provided in this study are included in the article. Further inquiries can be directed to the corresponding author.

## References

[1] Li B., Lai C., Zeng G., Huang D. (2022). Black phosphorus, a rising star 2D nanomaterial in the post-graphene era: Synthesis, properties, modifications, and photocatalysis applications. Small.

[2] Lin J., Ye W., Xie M., Seo D.H., Luo J., Wan Y., Bart V.B. (2023). Environmental impacts and remediation of dye-containing wastewater. Nat. Rev. Earth Environ..

[3] Asgharian M., Mehadipourghazi M., Khoshandam B., Keramati N. (2019). Photocatalytic degradation of methylene blue with synthesized rGO/ZnO/Cu. Chem. Phys. Lett..

[4] Motelica L., Oprea O.C., Vasile B.S., Ficai A., Ficai D., Andronescu E., Holban A.M. (2023). Antibacterial Activity of Solvothermal Obtained ZnO Nanoparticles with Different Morphology and Photocatalytic Activity against a Dye Mixture: Methylene Blue, Rhodamine B and Methyl OrangeJournal. Int. J. Mol. Sci..

[5] Dai C., Liu B. (2020). Conjugated polymers for visible-light-driven photocatalysis. Energy Environ. Sci..

[6] Wang S., Guan B., Lou X. (2018). Rction of ZnIn_2_S_4_-In_2_O_3_ hierarchical tubular heterostructures for efficient CO_2_ photoreduction. J. Am. Chem. Soc..

[7] Yang C., Xu H., Shi J., Liu Z., Zhao L. (2021). Preparation and Photocatalysis of CuO/Bentonite Based on Adsorption and Photocatalytic Activity. Materials.

[8] Mourya A.K., Singh R.P., Kumar T., Talmale A.S., Gaikwad G.S., Wankhade A.V. (2023). Tuning the morphologies of ZnO for enhanced photocatalytic activity. Inorg. Chem. Commun..

[9] Rabchinskii M.K., Sysoev V.V., Brzhezinskaya M., Solomatin M.A., Gabrelian V.S., Kirilenko D.A., Stolyarove D.Y., Saveliev S.D., Shvidchenko A.V., Cherviakova P.D. (2024). Rationalizing Graphene–ZnO Composites for Gas Sensing via Functionalization with Amines. Nanomaterials.

[10] Rabell G.O., Cruz M.A., Ramirez I.J. (2021). Hydrogen production of ZnO and ZnO/Ag films by photocatalysis and photoelectrocatalysis. Mater. Sci. Semicond. Process..

[11] Tan X., Zhou S., Tao H.J., Wang W.Y., Wan Q.W., Zhang K.C. (2019). Influence of Ag on photocatalytic performance of Ag/ZnO nanosheet photocatalysts. J. Cent. South Univ..

[12] Ahmad M., Ahmed E., Hong Z.L., Khalid N.R., Ahmed W., Elhissi A. (2013). Graphene-Ag/ZnO nanocomposites as high performance photocatalysts under visible light irradiation. J. Alloys Compd..

[13] Viet H.T.T., The H.C., Pham T.N., Pham T.T., Le M.C. (2019). Synergistic Adsorption and Photocatalytic Activity under Visible Irradiation Using Ag-ZnO/GO Nanoparticles Derived at Low Temperature. J. Chem..

[14] Tan L.L., Ong W.J., Chai S.P., Mohamed A.R. (2013). Reduced graphene oxide-TiO_2_ nanocomposite as a promising visiblelight-active photocatalyst for the conversion of carbon dioxide. Nanoscale Res. Lett..

[15] Motelica L., Vasile S., Fical A., Surdu A.V., Ficai D., Oprea O.C., Andronescu E., Jinga D.C., Holban A.M. (2022). Influence of the Alcohols on the ZnO Synthesis and Its Properties: The Photocatalytic and Antimicrobial Activities. Pharmaceutics.

[16] Gebretsadik A., Kafale B., Sori C., Tsegaye D., Murthy H.C.A., Abebe B. (2024). Cu-doped ZnO/Ag/CuO heterostructure: Superior photocatalysis and charge transfer. RSC Adv..

[17] Khan A., Shah A.A., Allehabi S.O., Ahmed F., Alsulami A., Azam A. (2024). Enhanced degradation of Ciprofloxacin (CIP) antibiotic and methylene blue (MB) dye using ZnO/GO nanocomposites under solar irradiation. Sci. Rep..

[18] Sharda P., Satendra K.C., Anchal S., Shukla R.K. (2024). Synergy of Fe dopant and graphite oxide in ZnO based nanocomposites for efficient photocatalytic degradation of indigo carmine dye. Ceram. Int..

[19] Rahman M.S., Suvo M.A.H., Islam M.M.T., Noor A.R., Yeachin N., Bhuiyan M. (2024). Fast and efficient removal of metronidazole from aqueous solution using graphene oxide (GO) supported nitrogen (N) doped zinc oxide (ZnO) nanoparticles. Colloid Surf. A..

[20] Abdullah S.A., Muhammad M.A., Alaa A.A., Mohamed M. (2024). Optimizing pollutant removal ability of GO/ZnO nanocomposites via controlled GO treatment and ZnO NPs content. Mater. Sci. Eng. B.

[21] Handan B., Durmus A., Colak H., Kurban R., Sahmetlioglu E., Karakose E. (2024). Investigation of the performance and properties of ZnO/GO double-layer supercapacitor. J. Phys. Chem. Solids.

[22] Wang P.Q., Bai Y., Luo P.Y., Liu J.Y. (2013). Graphene-WO_3_ nanobelt composite: Elevated conduction band toward photocatalytic reduction of CO_2_ into hydrocarbon fuels. Catal. Commun..

[23] Nasrollahzadeh M., Jaleh B., Jabbari A. (2014). Synthesis, characterization and catalytic activity of graphene oxide/ZnO nanocomposites. RSC Adv..

[24] Gao P., Ng K., Sun D. (2013). Sulfonated graphene oxide ZnO-Ag photocatalyst for fast photodegradation and disinfection under visible light. J. Hazard. Mater..

[25] Jaleh B., Jabbari A. (2014). Evaluation of reduced graphene oxide/ZnO effect on properties of PVDF nanocomposite films. Appl. Surf. Sci..

[26] Deepthi V., Anju S., Vidhya B. (2023). Influence of GO content on ZnO: GO composite thin films for visible light driven photocatalytic degradation of model pollutants. J. Sol-Gel Sci. Technol..

[27] Ravichandran K., Uma R., Sriram S., Balamurgan D. (2017). Fabrication of ZnO:Ag/GO composite thin films for enhanced photocatalytic activity. Ceram. Int..

[28] Doluel E.C., Kartal U., Dikici T., Yurddaskal M. (2020). Effect of Ag Content on Photocatalytic Activity of Ag@TiO_2_/rGO Hybrid Photocatalysts. J. Electron. Mater..

[29] Li G., Yang C., He Q., Liu J. (2022). Ag-based photocatalytic heterostructures: Construction and photocatalytic energy conversion application. J. Environ. Chem. Eng..

[30] Fan Z., Li C., Xu M. (2024). Fabrication of ZnO/Ag photocatalyst and its photocatalytic degradation properties on trimethylamine. J. Iran. Chem. Soc..

[31] Feng C., Chen Z., Jing J., Hou J. (2020). The photocatalytic phenol degradation mechanism of Ag-modified ZnO nanorods. J. Mater. Chem. C..

[32] Mishakav I.V., Bauman Y.I., Brzhezinskaya M., Netskina O.V., Shubin Y.V., Kibis L.S., Stoyanovskii V.O., Larionov K.B., Serkova A.N., Vedyagin A.A. (2022). Water purification from chlorobenzenes using heteroatom-functionalized carbon nanofibers produced on self-organizing Ni-Pd catalyst. J. Environ. Chem. Eng..

[33] Sobaszek M., Brzhezinskaya M., Olejnik A., Mortet V., Alam M., Sawczak M., Ficek M., Gazda M., Weiss Z., Bogdanowica R. (2023). Highly Occupied Surface States at Deuterium-Grown Boron-Doped Diamond Interfaces for Efficient Photoelectrochemistry. Small.

[34] Rabchinskii M.K., Ryzhkov S.A., Besedina N.A., Brzhezinskaya M., Malkov M.N., Stolyarova D.Y., Arutyunyan A.F., Struchkov N.S., Saveliev S.D., Diankin I.D. (2022). Guiding graphene derivatization for covalent immobilization of aptamers. Carbon.

